# Mindfulness-Based Cognitive Therapy Experiences in Youth With Inflammatory Bowel Disease and Depression: Protocol for a Mixed Methods Qualitative Study

**DOI:** 10.2196/14432

**Published:** 2019-07-24

**Authors:** Tatjana Ewais, Jakob Begun, Maura Kenny, Alan Headey, Steve Kisely

**Affiliations:** 1 Mater Clinical School and Princess Alexandra Clinical School School of Medicine University of Queensland South Brisbane Australia; 2 Mater Young Adult Health Centre Mater Misericordiae Ltd South Brisbane Australia; 3 Faculty of Health and Medical Sciences University of Adelaide Adelaide Australia

**Keywords:** mindfulness, inflammatory bowel disease, qualitative research, adolescents, young adults

## Abstract

**Background:**

Mindfulness-based programs are increasingly used as a part of integrated treatment for inflammatory bowel disease (IBD). However, the majority of research has been quantitative with limited qualitative exploration of patients’ experiences of mindfulness programs and no studies among adolescents and young adults with IBD. Furthermore, there has been a paucity of research exploring the role of common psychotherapy and group factors within mindfulness programs.

**Objective:**

This study aims to explore the experiences of adolescents and young adults with IBD and depression who completed a mindfulness-based cognitive therapy (MBCT) group program, as well as the role of therapeutic alliance, group affiliation, and other common psychotherapy and group factors.

**Methods:**

This mixed methods qualitative study, nested within a randomized controlled trial (RCT) of MBCT for adolescents and young adults with IBD, will obtain qualitative data from focus groups and open-ended survey questions. The study aims to conduct three to four focus groups with 6-8 participants in each group. It will employ data and investigator triangulation as well as thematic analysis of the qualitative data.

**Results:**

The study was approved by the Mater Hospital Human Research Ethics Committee and recruitment commenced in May 2019; study completion is anticipated by early 2020.

**Conclusions:**

The study will contribute to the assessment of acceptability and feasibility of the MBCT program for adolescents and young adults with IBD. It will also elucidate the role of previously unexplored common psychotherapy and group factors within mindfulness training and help inform the design of a future large-scale RCT of MBCT in this cohort.

**Trial Registration:**

Australian New Zealand Clinical Trials Registry (ANZCTR) ACTRN12617000876392; https://www.anzctr.org.au/Trial/Registration/TrialReview.aspx?id=373115

**International Registered Report Identifier (IRRID):**

PRR1-10.2196/14432

## Introduction

### Background

Inflammatory bowel disease (IBD) is an immune-mediated condition characterized by chronic inflammation of the gastrointestinal tract, a relapsing and remitting course, and frequent systemic manifestations [1-3]. The peak age of onset is between the ages of 15 and 29, with wide-ranging implications in all areas of life, including relationships, education, and employment opportunities [4,5]. As a result of the high burden of illness in IBD, adolescents and young adults (AYAs) with IBD experience disruption at a crucial developmental stage with significantly impaired quality of life as well as rates of depression and anxiety two to three times higher than in the general population or among youth with other chronic diseases [6,7]. There is well-documented research evidence supporting the impact of depression on the course of IBD as well as the bidirectional relationship between IBD and depression, showing that they can both precipitate the onset and worsen the course of each other [8].

### Mindfulness, Inflammatory Bowel Disease, and Depression

Mindfulness interventions are defined as therapeutic interventions based on core mindfulness principles; they contain various informal and formal practices, such as mindfulness of the breath, body scanning, mindful movement or yoga, and open or choiceless awareness. Mindfulness as a concept is described as a process of nonjudgmental, intentional awareness of one’s internal and external reality, characterized by “paying attention in a particular way: on purpose, in the present moment and non-judgmentally” [9]. Mindfulness-based interventions have been increasingly trialed in IBD patients because of their potential to treat both depression and IBD [10,11], as well as attenuating immune system abnormalities, thereby improving the course of IBD [12,13]. Mindfulness-based stress reduction (MBSR) and mindfulness-based cognitive therapy (MBCT) are two manualized, 8-week group programs with strong evidence in the treatment of depression and anxiety that have been used successfully in integrated treatment of individuals with IBD [14-17]. The mindfulness-based cognitive therapy program used in this study is an 8-week group mindfulness program with weekly sessions of 2 hours, which contain a mixture of various cognitive skills and mindfulness practices. These are taught and practiced during the sessions as well as between sessions in home practices. The program closely follows the original MBCT curriculum designed by Segal, Williams, and Teasdale [18], adapted for both IBD and young adults. Modifications related to IBD include IBD-specific teaching content, such the role of stress in flares and gut-brain axis, as well as modified mindful movement practices with more restorative yoga and adapted postures for those with joint pain and perianal disease. Developmental modifications include shortened mindfulness practices, as AYAs often cannot sustain longer practices compared to adults; modified mindful movement practices, as AYAs often prefer mindful movement and yoga to body scanning and often prefer a slightly faster pace; fun postcards to help identify emotional states; and youth-friendly poetry.

Although mindfulness-based treatments have been used successfully in adults with IBD [14-17] and among AYAs with other chronic illnesses and depression [19-21], there have been no studies of mindfulness programs in AYAs with IBD. The majority of research has involved quantitative clinical trials investigating the impact of mindfulness training on psychosocial and disease-related parameters in individuals with IBD, with only one qualitative study exploring adult IBD sufferers’ experiences of a mindfulness-based program [22]. Importantly, there have been no studies investigating mindfulness in AYAs with IBD.

We will therefore conduct a mixed methods qualitative study to explore experiences of MBCT in AYAs with IBD who are currently participating in the randomized controlled trial (RCT) of an IBD-focused and developmentally informed MBCT program; we will also explore the role of common psychotherapy and group factors in the MBCT program. The study will collect and analyze qualitative data from two different sources: focus groups and open-ended questions from the post-MBCT evaluation survey.

### Common Psychotherapy Factors and Group Factors in Mindfulness Training

Common factors in psychotherapy are therapeutic elements that are common to diverse psychotherapies and are responsible for many of their therapeutic benefits [23,24]. These factors may account for relative therapeutic equivalence of outcomes for a range of psychotherapeutic models that are found in meta-analytic studies [23-29]. The notion of common factors was first introduced in 1936 in Rosenzweig’s seminal paper, which outlined the therapeutic relationship, therapy rationale or ideology, and integration of subsystems of the patient’s personality and the therapist’s personality as key common factors present in all types of psychotherapies [23]. Common factors were subsequently expanded to include delivery of prescribed treatments or *rituals* [30] and *enactment* of adaptive or health-promoting actions [24]; factors were grouped into nonspecific common factors, such therapeutic alliance and expectations, and specific factors, such as exposure and sense of mastery [25,27]. It is likely that common psychotherapy factors operate within mindfulness programs as they, in addition to mindfulness skills training, contain reflective, exploratory, and supportive elements; mindfulness itself has been postulated as one of the core common factors in psychotherapy [31]. Despite this, there has been only one study to date exploring the role of common factors in mindfulness training [32] and no studies exploring group factors in mindfulness interventions.

Group factors are key psychotherapeutic factors that facilitate change in group therapies and include the following: instillation of hope, universality, imparting information, altruism or helping others in the group, corrective recapitulation of the primary family group through developing connections within the group, development of socializing through group communication, interpersonal learning, group cohesiveness or social affiliation, experience of relief associated with free emotional expression, and existential factors [33]. Although these group factors were initially considered specific to psychotherapeutic groups, it has since been accepted that they are present in most group settings, including education and support groups [34,35], and they are likely to have a role within mindfulness groups.

Our decision to explore the role of common psychotherapy and group factors in mindfulness training was driven by an informed hypothesis of their potential role in mindfulness-based therapies, as well as consistent and recurrent feedback from MBCT group participants that the most important factors in their recovery were *mindfulness* and *friendship*. Many of the reported benefits were related to the common factors of exposure, mastery, and expectations. They also commented on their perceived positive impact of the therapy, the facilitator’s skills, and their engagement to the group facilitator, which were consistent with common factors of therapeutic alliance and the therapist’s personality. Their description of *friendship*, which they rated as the most important of all MBCT benefits, focused on a sense of belonging, social affiliation, and peer support. These experiences of *friendship* or *belonging* are in stark contrast to their pre-MBCT experience of feeling isolated with a chronic illness. Their description of *friendship* created within and outside of the MBCT group corresponded to the therapeutic group factors of social affiliation and engendering hope.

### Objectives

The purpose of this study is to explore AYAs’ experiences of a developmentally informed and IBD-focused MBCT group with a focus on their views of the program’s benefits and acceptability, perceived barriers, and suggestions for further adaptation. A secondary objective is to investigate the role of therapeutic alliance, group affiliation, and other common psychotherapy and group factors within mindfulness training.

## Methods

### Study Design

This is a mixed methods qualitative study exploring experiences of AYAs with IBD participating in an MBCT program. This qualitative study is embedded within the RCT of an adapted MBCT program for AYAs with IBD and depression, which is described in detail in the study protocol [36]. The design adheres to the consolidated criteria for reporting qualitative research (COREQ) guidelines, a 32-item checklist for interviews and focus groups [37]. The study will use two different sources of qualitative data, including focus groups and free-text questions from the post-MBCT evaluation survey. Focus groups will be conducted and analyzed according to Krueger and Casey’s focus groups guide [38]. An inductive thematic analysis approach will be used to analyze the qualitative data [39].

### Rationale for Employing a Mixed Methods Qualitative Approach

Mixed methods research is defined as the use of different methodological approaches in a single study or a set of related studies, which are likely to create more meaningful and ultimately more useful data in answering the research questions [40]. In recent years, the concept of mixed methods research has been expanded to any research that combines different styles of research, not restricted to combining quantitative and qualitative methods. Mixed methods research is also known as “between research paradigm mixing” but may also include “within research paradigm mixing.” The latter can refer to mixing different qualitative approaches, such as qualitative interviews, participant observation, and qualitative documents, as well as mixing different quantitative approaches, such as quantitative surveys combined with quantitative experimental research [40,41].

We chose a mixed methods approach combining qualitative data from different sources, as previous research in this area has supported the use of focus groups in combination with surveys [22]. Focus groups also fit the study’s dual purpose of evaluating the MBCT program through participants’ views of its feasibility and acceptability, while exploring their experiences and views of common therapeutic and group factors within the mindfulness group. They are particularly well suited to understanding MBCT group experiences, given their permissive and inclusive group processes and nondirective interviewing techniques that facilitate expression of participants’ views, attitudes, verbal and nonverbal interactions, and shared experiences [38,42]. Furthermore, focus groups may hold an advantage over individual interviews in that they can provide additional information through promoting group exchanges and self-disclosure in this cohort of young participants with similar backgrounds, as participants “tend to disclose more of themselves to those who resemble them in various ways” [38].

Combining qualitative data from focus groups and MBCT evaluation surveys, as well as using two researchers for coding and analysis, will ensure a rich dataset and facilitate data and investigator triangulation and thematic saturation, thus strengthening the study validity.

### Recruitment and Sampling

Participants will be recruited from the 64 participants enrolled in the RCT of MBCT for AYAs with IBD and depression. Inclusion criteria will be participation in the RCT of MBCT for AYAs with IBD and completion of the MBCT course, defined as attendance at a minimum of five out of eight sessions of the program, the accepted benchmark for completion. We will also record the reasons for nonengagement among those who decline participation or drop out before completion; these will be collated and discussed in the final study report.

All the participants who completed the MBCT group program will be asked to complete a post-MBCT evaluation survey, which contains qualitative questions about their group experiences. They will also be invited to participate in the focus groups. Participants will be offered parking vouchers and public transport cards; focus groups will be conducted after hours to accommodate those studying or working.

As there will be four MBCT group programs conducted over 2 years, participants will provide their post-MBCT evaluation surveys upon completion of the MBCT group program; this qualitative data will be collated and analyzed following the last MBCT group program completion. Participants will also be invited to participate in focus groups upon completing the MBCT program and after completing the MBCT evaluation survey. The recruitment for focus groups commenced following the second MBCT group program completion in May 2019 to ensure sufficient focus group participant numbers. Focus groups will be conducted between June and December 2019. Participants will be invited to participate in focus groups via face-to-face meetings, email, and phone, and those who express interest will meet with the research assistant who will explain the focus group process in more detail and will obtain consent.

Sampling will be purposive, in keeping with the definition of purposive sampling from the COREQ guidelines, which state that this “involves selecting participants who share particular characteristics and have the potential to provide rich, relevant and diverse data pertinent to the research question” [37]. Purposive sampling selects participants based on the study purpose, in contrast with convenience sampling, which selects participants based on “certain practical criteria, such as easy accessibility, geographical proximity, availability at a given time, or the willingness to participate” [43]. We are therefore selecting all participants who completed the MBCT program, as this is a characteristic relevant to our study purpose of exploring the experience of MBCT in AYAs who completed the program. Specifically, we will invite all the participants who completed the MBCT program to complete the MBCT evaluation survey and to participate in focus groups. The sampling for the focus groups will also be consecutive; it will proceed until the desired number of participants have been recruited to conduct a sufficient number of focus groups in order to achieve thematic saturation. We envisage running three to four focus groups of 6-8 participants in each. This size of focus group is consistent with the recommended number of participants for clinical focus groups to accommodate for the clinical nature of the group; facilitate moderation and expression of individual opinions; and provide rich, but not overwhelming, data [39,44,45]. Therefore, our proposed group size of 6-8 participants is in keeping with these generally accepted recommendations and fits the nature of our clinical sample. The recommended number of focus groups varies depending on the purpose of the research, group homogeneity, and use of additional sources of qualitative data, with most guides proposing that three to four focus groups are likely to be sufficient to achieve across-group analysis and thematic saturation [38,44,45]. Our proposed number of focus groups and their size is consistent with these recommendations and, therefore, likely to provide us with a sufficient sample size to achieve thematic saturation.

### Development of Questioning Route for Focus Groups

We followed Krueger and Casey’s recommendations in developing the questioning route by creating questions that are clear, engaging, and likely to evoke conversation among the participants [38]. Our questioning route contains a mixture of open-ended and more targeted questions about participants’ group experiences. We also created questions regarding their views on the role of common psychotherapeutic factors (eg, engagement with and the role of the group facilitator) and affiliation with peers in the same age group with the same medical condition. The questioning route for focus groups is summarized in Textbox 1.

**Questioning route for inflammatory bowel disease (IBD) focus groups.**

Purpose: Thank you for coming. The purpose of this group is to understand your experience of participating in the IBD mindfulness study. We want to know what it was like for you. There are definitely no “right” or “wrong” answers. It’s all about how you found it. Please feel free to share your point of view even if it differs from what others have said.

We will be recording today’s session so that we can transcribe it later. All names and any identifying information will be removed from any publications. You won’t be identified in any way.

You can take a break at any stage, please just let me know.

Does anybody have any questions? OK, well let’s go...

 

Question 1. To start with, can you say who you are and when you started practicing mindfulness/completed the MBCT group.

Question 2. What was the group like for you?

Question 3. What was it like being in a group with other young people that live with inflammatory bowel disease (IBD)?

Question 4. How did you find the facilitator?

Question 5. Which components of the MBCT program did you find the most/least beneficial and why (ie, body scanning, mindfulness of the breath, mindful movement, homework, or teaching)?

Question 6. If there is one thing you’re taking away from this group, what’s that?

Question 7. Let’s say you never did this group. Where would you be now?

Question 8. Would you recommend this group to someone else?

– What if they said, “I’m too nervous”?

Question 9. Is there anything you did not like, or that you think we should do differently?

Question 10. All things considered, of all issues that we’ve discussed today, what do you consider to be the most important?

Question 11. Have we missed anything?

Question 12. Summary question (after a brief oral summary): Is this an adequate summary?

Focus groups will be conducted in a facility purposefully designed for focus group interviewing, with equipment for audiotaping and a viewing room allowing observers to unobtrusively watch the interviews through a one-way mirror. Each focus group will last from 1 to 2 hours. Focus groups will be run by the moderator and an assistant, with an observer behind the one-way mirror. The observer and the assistant will observe participants’ verbal and nonverbal interactions that may be missed by the moderator who is immersed in running the focus group; they will also take notes of relevant quotes, key points, ideas, and themes.

The moderator (AH) is a psychologist whom most of the participants have not met prior to the focus groups. The assistant is the study research assistant with a degree in public health, and the observer (TE) is the study principal investigator who is a psychiatrist by training. Participants will meet the research assistant and the observer during the RCT recruitment.

The moderator will follow the questioning route guide and facilitate discussion relevant to the study purpose. As soon as the focus group is finished, the moderator, assistant, and observer will meet outside the room to discuss any additional observations and decide on asking any follow-up questions. For instance, it may be that the observer or assistant notice that a participant was interrupted while raising an interesting point. In such cases, the facilitator will return and ask a follow-up question. These additional observations and questions will be included in the follow-up research team meetings and potentially provide a richer dataset.

Participants will be given the opportunity to review focus group transcripts. They will be provided with the results of preliminary analysis and given the opportunity to comment. At the end of the study, a summary of the study findings will be given to all participants.

### Mindfulness-Based Cognitive Therapy Evaluation Survey

Upon conclusion of the 8-week MBCT program, all participants who completed the course will be asked to fill out the post-MBCT evaluation survey (see Multimedia Appendix 1). The survey contains a mixture of closed-ended and open-ended free-text questions exploring participants’ experiences, expectations, perceived barriers, and benefits, as well as suggestions for program improvement. Surveys are a commonly used method in health research, and qualitative analysis of free-text open-ended survey questions has been recognized as an important tool in providing valuable insights into participants’ views and experiences [46,47]. Open-ended survey questions complement the focus group questions and combining their qualitative data in thematic analysis will provide a richer dataset and strengthen the study validity.

### Data Analysis

Focus groups will be audiotaped and transcribed verbatim. Qualitative data from focus groups and open-ended survey questions will be analyzed using thematic analysis [39]. Thematic analysis has been chosen because it is a flexible qualitative research method for identification, analysis, and reporting of key themes and subthemes within data that can highlight similarities and differences within the data and generate insights [39]. Furthermore, an inductive thematic analysis approach will suit the exploratory nature of our study.

Analysis will start with two research team members—moderator and observer—who will first familiarize themselves with the data through reading and rereading of the collected data. They will individually and separately develop initial codes based on emerging clusters of statements. The coding process will follow the recommendations outlined in Saldaña’s Coding Manual for Qualitative Researchers [47] in that the researchers will develop and test the codes in two cycles. Initial codes will be developed in the first coding cycle and tested on the subsection of data to eliminate any overlapping codes, refine the existing codes, and introduce any new codes. The second coding cycle will further refine and highlight salient features of the codes to facilitate subsequent generation of categories, concepts, and themes. The final codes will be independently applied to the full dataset by the two researchers and discussed until an agreement is reached on the interpretation. The analysis and grouping of the codes will enable identification of categories, followed by analysis and comparing of the major categories, leading to emergence of relevant themes and concepts.

The researchers will work iteratively and inductively with the data until distinct themes develop and no new themes emerge (ie, thematic saturation is achieved). The emerging themes will be discussed among the investigators to achieve triangulation and ensure that all relevant data are captured. Further triangulation will be achieved by combining different sources of data: focus groups transcripts and open-ended free-text qualitative questions from the post-MBCT evaluation survey. Representative quotes will be used to illustrate the codes and themes.

## Results

This study was funded by the Brain-Injured Children’s Aftercare Recovery Endeavours (BICARE) project grant in January 2018 and approved by the Mater Hospital Human Research Ethics Committee. Recruitment commenced in May 2019; completion of the qualitative data analysis and results are anticipated by early 2020.

## Discussion

There is a paucity of qualitative studies investigating mindfulness program participants’ experiences among the IBD population, despite a multitude of quantitative trials exploring the efficacy of mindfulness programs in treating IBD-related psychosocial comorbidities and their impact on the course of IBD. The only study to date of MBCT experiences among individuals with IBD was conducted in adults and focused predominantly on participants’ views of the MBCT program’s barriers and benefits [22]. Furthermore, there has been only one study to date investigating the role of common factors in mindfulness interventions [32] and no studies exploring the role of group factors in mindfulness-based interventions.

To our knowledge, this mixed methods qualitative study will be the first to use a more inductive approach to explore participants’ MBCT experiences and the only study conducted in AYAs with IBD. This study will also be the first to investigate the role of therapeutic alliance, sense of mastery, group affiliation, and other common psychotherapy and group factors within mindfulness training. It will employ thematic analysis of qualitative data from focus groups and open-ended qualitative survey questions. The study will fulfil the dual purpose of exploring the experiences, feasibility, and acceptability of the MBCT program among AYAs with IBD, as well as providing greater understanding of the role of common psychotherapeutic and group factors within the mindfulness program.

The study findings will facilitate interpretation of the results of the RCT of MBCT in AYAs with IBD and will help inform the design of a future large RCT of MBCT in this patient cohort.

## Appendix

Multimedia Appendix 1 The mindfulness-based cognitive therapy group evaluation survey.

## Abbreviations

AYAs: adolescents and young adults
BICARE: Brain-Injured Children’s Aftercare Recovery Endeavours
COREQ: consolidated criteria for reporting qualitative research
IBD: inflammatory bowel disease
MBCT: mindfulness-based cognitive therapy
MBSR: mindfulness-based stress reduction
RCT: randomized controlled trial
