# Supplementary material for: Mindfulness-Based Cognitive Therapy Experiences in Youth With Inflammatory Bowel Disease and Depression: Protocol for a Mixed Methods Qualitative Study
Source: JMIR Res Protoc. 2019 Jul 24;8(7):e14432. doi: 10.2196/14432 (PMC6685121; doi:10.2196/14432)
Supplement: Multimedia Appendix 1 [file resprot_v8i7e14432_app1.docx]

**Appendix A: The Mindfulness Based Cognitive Therapy Group Evaluation Survey**

Indicate your level of agreement or disagreement with the following statements, by circling the number that best reflects your opinion

| *#* | Question |
| --- | --- |
| 1 | My initial reason for taking the course has been addressed by way of the practices and/or the group enquiry & discussion.  (Strongly disagree) 1………….2………….....3………….. .4……………. 5…………. 6 (Strongly agree) |
| 2 | I have learned some tools that are useful to me.  (Strongly disagree) 1………….2………….....3…………... 4………………5……………6 (Strongly agree) |
| 3 | Making time for homework was difficult but became easier in time.  (Strongly disagree) 1………….2………….....3…………... 4……………….5………… 6 (Strongly agree*)* |
| 4 | I was able to uncover and observe my own obstacles /resistance to practice.  (Strongly disagree) 1………….2………….....3…………... 4……………… 5………… 6 (Strongly agree*)* |
| 5 | What did you want or hope for in attending the course? |
| 7 | I learned that meditation is not about chasing away thoughts, or controlling my thoughts, or to have “better” thoughts.  (Strongly disagree) 1………….2………….....3………….…4………………5……………6 (Strongly agree) |
| 8 | The content about stress physiology was interesting and informative  (Strongly disagree) 1………….2………….....3…………... 4……………….5………….. 6 (Strongly agree)  If you disagree, please state why: |
| 9 | The group content on IBD and self-management was of value  (Strongly disagree) 1………….2………….....3…………... 4………………. 5…………...6 (Strongly agree)  If you disagree, please state why: |
| 10 | I feel confident about being able to manage my health condition using mindfulness skills  (Strongly disagree) 1………… 2………….....3………….. 4……………… 5……………6 (Strongly agree)  Comment if disagree: |
| 11 | Emotional issues arising during the practice were manageable with the tools I learned in class…or I felt free to contact the instructor  (Strongly disagree) 1………….2…………....3…………... 4……………….5……………6 (Strongly agree) |
| 12 | The pace of the course was suitable.  (Strongly disagree) 1………….2………….....3…………...4……………….5……………6 (Strongly agree)  More time could have been spent on: |
| 13 | The instructor’s style suited the course material.  (Strongly disagree) 1………….2………….....3…………...4……………….5…………….6 (Strongly agree)  If not, why not? What can be improved? |
| 14 | The materials (handouts & voice tracks) were clear and helpful.  (Strongly disagree) 1………….2………….....3…………...4……………….5…………….6 (Strongly agree) |
| 15 | What were the obstacles/costs to you? |
| 16 | What may help you in the future if you are in danger of becoming overwhelmed? |
| 17 | How important the course has been and why? |
| 18 | Any other feedback for the group facilitator? |
